# Effects of guanidino acetic acid and betaine supplementation on growth, dietary nutrient digestion and intestinal creatine metabolism in sheep

**DOI:** 10.1002/vms3.1470

**Published:** 2024-06-24

**Authors:** Chen Ma, Mireguli Yimamu, Shiqi Zhang, Ali Mujtaba Shah, Hao Yang, Wenjie Cai, Chaonan Li, Xuejie Lu, Fengming Li, Kailun Yang

**Affiliations:** ^1^ College of Animal Science Xinjiang Agricultural University Urumchi China; ^2^ Key Laboratory of Animal Genetics Breeding and Reproduction of Shaanxi Province College of Animal Science and Technology Northwest A&F University Xianyang China

**Keywords:** betaine, creatine metabolism, guanidino acetic acid, sheep, transcriptomics

## Abstract

**Background:**

The intestine of young ruminants is in the developmental stage and has weaker resistance to the changes of external environment. Improving intestinal health is vital to promoting growth of young ruminants. This study investigated effects of guanidino acetic acid (GAA) and rumen‐protected betaine (RPB) supplementation on growth, dietary nutrient digestion and GAA metabolism in the small intestine of sheep.

**Methods:**

Eighteen healthy Kazakh rams (27.46 ± 0.10 kg of body weight and 3‐month old) were categorized into control, test group I and test group II, which were fed a basal diet, 1500 mg/kg GAA and 1500 mg/kg GAA + 600 mg/kg RPB, respectively.

**Results:**

Compared with control group, test group II had increased (*p* < 0.05) average daily gain, plasma creatine level, ether extract (EE) and phosphorus digestibility on day 30. On day 60, the EE apparent digestibility, jugular venous plasma GAA, GAA content in the duodenal mucosa and GAA content in the jejunal and ileal mucosa of test group II were higher (*p* < 0.05) than other groups. Transcriptome analysis revealed that the differentially expressed genes (DEGs) involved in the duodenal pathways of oxidative phosphorylation and non‐alcoholic fatty liver disease were significantly altered in test group II versus test group I (*p* < 0.05). Moreover, in the jejunum, the MAPK signalling pathway, complement and coagulation cascade and B‐cell receptor signalling pathway were significantly enriched, with ATPase, solute carrier transporter protein, DHFR, SI, GCK, ACACA and FASN being the significantly DEGs (*p* < 0.05).

**Conclusion:**

Dietary supplementation of RPB on top of GAA in sheep diets may promote sheep growth and development by improving the body's energy, amino acid, glucose and lipid metabolism capacity.

## Graphical abstract

Dietary supplementation of rumen‐protected betaine on top of guanidino acetic acid in sheep diets can improve the apparent digestibility of ether extract, phosphorus and creatine levels in small intestinal mucosal tissues. Additionally, supplemental feeding of rumen‐protected betaine and guanidino acetic acid may promote growth and development of sheep by improving the body's energy, amino acid, glucose and lipid metabolism capacity.

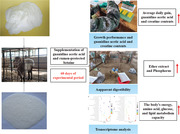

## 1 INTRODUCTION

In vertebrates, guanidino acetic acid (GAA) is the only precursor of creatine and is produced by the combination of l‐arginine and glycine under the action of arginine: glycine amidinotransferase. Briefly, GAA is translocated from the kidneys to the liver through blood. In the liver, the methyl group of *S*‐adenosylmethionine (SAM) is transferred to GAA under the action of guanidinoacetate amidino transferase to synthesize creatine (Negro et al., 2019).

Creatine is a nitrogenous amino acid that performs crucial functions in animals such as promoting growth and improving muscle energy supply (Zhang et al., 2014). Additionally, it plays a key role in the adenosine triphosphate (ATP) cycle by generating phosphocreatine following the action of creatine kinase. In vertebrate cells, creatine and phosphocreatine are the key substrates for energy transfer, and the phosphocreatine and glycogen contents in muscles can be increased via the phosphocreatine–creatine energy supply system (Farshidfar et al., 2017). Specifically, creatine synthesis, carnitine, phosphatidylcholine synthesis, epinephrine synthesis and DNA methylation require dietary or endogenously synthesized methyl. In addition, all methylation reactions play an important role in growth and development by maintaining the basic functions of the organism as well as ensuring continuous accumulation to meet the needs of growth and performance (Ostojic et al., 2014). Betaine, a derivative of glycine metabolism in animals and plants, can be used to replace choline and methionine as a source of methyl, thereby reducing feed cost incurred by choline and methionine supplementation (Nutautait et al., 2020). Moreover, during creatine synthesis in animal production, exogenously supplied GAA consumes large amounts of methyl, which can be sourced from methionine, betaine, choline and folic acid (Ardalan et al., 2020; Chandani et al., 2021; Degroot et al., 2019).

It is well known that the intestine is the main site of nutrient digestion and absorption, as well as the regulation of microflora and immune function (Macia et al., 2015). In addition, the intestine is involved in regulating metabolism and processes such as endocrine function and is a key organ in promoting animal health and productivity (Chambers et al., 2015). A previous study found that dietary supplementation of GAA reduced the mRNA expressions of interleukin‐1β and tumour necrosis factor‐α, which had positive influence in attenuating inflammatory response of small intestine caused by heat stress and improving intestinal morphology (Peng et al., 2023). In addition, a recent study reported that betaine supplementation enhanced the barrier function of small intestine by increasing the mRNA expressions of junctional adhesion molecules‐B, occludin and zonula occludens‐1 (Won et al., 2023). Most of the available evidences on the digestive tract of sheep have focused on the anatomy, physiology and nutrition absorption mechanisms of the gastrointestinal villi, immune barrier and microflora; however, only a few studies have focused on the intestinal transcriptome. Foote et al. (2017) studied the transcriptomic response to isobutyl butyrate injected into the duodenum of lambs using RNA sequencing, and results revealed glycolysis, fatty acid activation/biosynthesis and up‐regulation of genes related to mitochondrial activity. Accordingly, the present study investigated the effect of rumen‐protected betaine (RPB) and GAA supplementation on body weight (BW), average daily gain (ADG), dry matter intake (DMI) and nutrient digestibility in sheep. Moreover, the study evaluated the effect of GAA and RPB supplementation on creatine metabolism in the small intestine of sheep. For this, transcriptomics analysis was used to sequence the duodenal and jejunal tissues from sheep receiving supplementation to screen out the relevant differentially expressed genes (DEGs) and related pathways.

## 2 MATERIALS AND METHODS

### 2.1 Experimental materials

GAA, without rumen‐protection treatment, (98% purity) was purchased from Genentech Biotechnology Co., Ltd. RPB, with a rumen rate of >80% and effective content of ≥40%, was purchased from Jiangxi Yuanchang Industry Co., Ltd.

### 2.2 Experimental design

Eighteen healthy Kazakh rams (27.46 ± 0.10 kg of BW and 3‐month old) were divided into the control group, test group I and test group II (*n* = 6 each) based on BW. All groups underwent a pre‐feeding period of 15 days, followed by a regular feeding period of 60 days. The doses of supplements were based on those reported by Bampidis et al. (2022). The control group was fed the basal diet, test group I was supplemented with 1500 mg/kg GAA, and test group II was supplemented with 1500 mg/kg GAA + 600 mg/kg RPB. The nutritional composition of the basal diet is shown in Table 1.

**TABLE 1:** Nutritional composition of the basal diet (DM basis).

Ingredients	Proportion (%)	Nutrient^b^	Content (%)
Alfalfa	20	DM	91.47
Wheat grass	20	ME (MJ/kg)	7.92
Corn	30	OM	89.52
Wheat bran	7.2	CP	14.62
Soybean meal	12	EE	1.95
Cottonseed meal	7.8	NDF	46.16
Premix^a^	3	ADF	32.62
Total	100	Calcium Phosphorus	0.52 0.37

Abbreviations: ADF, acid detergent fibre; CP, crude protein; DM, dry matter; EE, ether extract; ME, metabolic energy; NDF, neutral detergent fiber; OM, organic matter.

^a^ The premix provided the following nutrients and minerals (per kg): VA 2550 IU, VD3 2550 IU, VE 200 IU, niacin 20 mg, biotin 0.06 mg, Cu 22 mg, Fe 94 mg, Mn 80 mg, Zn 88 mg, I 0.75 mg, Se 0.5 mg, Co 0.33 mg, calcium 0.35%, phosphorus 0.125% and NaCl 0.8%.

^b^ Metabolic energy is indicated as the calculated value, and all other nutrients are indicated as measured values.

### 2.3 Breeding management

The test sheep were numbered and housed in a semi‐open and well‐ventilated sheep barn (1.2 m × 1.5 m). After the pre‐testing period, animals were fed twice daily at 08:00 and 20:00. Briefly, GAA and RPB were thoroughly mixed with 50 g of the supplement concentrate and were provided before feeding the daily diet. After this, 40% of the roughage and 60% of the concentrate supplement were fed. The amount of leftover feed was ensured to be <5% after free feeding by weighing it before the morning feeding. All animals were weighed every 30 days to calculate the average DMI, ADG and feed conversion ratio (FCR). In addition, all animals underwent disinfection, deworming and immunization according to routine sheep farm procedures.

### 2.4 Sample collection and processing

#### 2.4.1 Blood samples

For all sheep, blood was collected from the jugular vein at 0 h before morning feeding on days 30 and 60 of the test period. The obtained sample was placed in a sodium heparin anticoagulated blood collection tube and centrifuged at 3500 rpm for 15 min to separate the plasma. The supernatant plasma obtained was then divided into three parts by transferring to 2.0 mL Eppendorf tubes were stored at −20°C until further analysis. In addition, on day 60, the sheep were administered intramuscular xylazine hydrochloride into the neck 3.5 h after morning feeding. At 4 h after feeding, blood was collected in vivo from the superior mesenteric vein using a blood collection vessel containing sodium heparin anticoagulant. The obtained blood was centrifuged at 3500 rpm for 10 min to separate plasma, which was frozen at −20°C until further analysis.

#### 2.4.2 Faecal samples

The daily faecal output was calculated using the total collection method (Baleseng et al., 2023). Briefly, on days 27–30 and 55–58 of the experiment, faecal samples of all sheep were collected four times a day for 4 consecutive days. The samples were mixed for 4 consecutive days, and 10% of the total amount was randomly weighed, naturally dried, and then weighed. Finally, the faecal samples were sieved through a 40 mesh and preserved for the determination of nutrient content.

#### 2.4.3 Tissue samples

After plasma collection, six sheep of each group were bled to death under anaesthesia, and the ends of the junctions of the rumen, duodenum, jejunum, ileum and cecum were quickly ligated using 12‐ga sutures to intercept the duodenum and jejunum. After collecting each section of the small intestine, the chyme was tied tightly at one end, and the other end was washed with saline five times to clean the chyme residue. The small intestine was then dissected longitudinally from one end, and the intestinal section was wiped with filter paper to absorb the residual water on the surface of the intestinal mucosa. Subsequently, the surface mucosa of the duodenum and jejunum was scraped using a slide and mixed it separately. Then, the samples were divided into sterile, RNA‐degrading enzyme‐free lyophilization tubes and immediately stored in liquid nitrogen until further analysis. The sampling process was finished within 20 min to ensure the samples that can be used to do the following analysis.

### 2.5 Measurement of indicators

#### 2.5.1 Measurement of growth performance

On days 1, 30 and 60, the BW of all sheep were determined before morning feeding at 07:00. Subsequently, ADG was calculated, and DMI was recorded; based on these values, FCR was calculated by dividing DMI by ADG.

#### 2.5.2 Determination of plasma GAA metabolites

Plasma GAA, creatine and creatinine levels were determined as reported by Wada et al. (2012). Briefly, 200 μL of the supernatant was transferred to another Eppendorf tube. To this, 400 μL of acetonitrile was added and mixed. The mixture was then allowed to stand for 10 min to precipitate proteins and then centrifuged at 12,000 rpm for 10 min. Subsequently, 100 μL of the supernatant was mixed with 200 μL of phosphoric acid (2 mM). From this, a 20‐μL sample was assessed using high‐performance liquid chromatography using the IC YS‐50 weakly acidic cation‐exchange column (4.6 mm × 125 mm). Chromatographic conditions were as follows: flow rate, 1.0 mL/min; column temperature, 30°C; detection wavelength, 210 nm; elution mode, linear elution; injection volume, 20 μL.

#### 2.5.3 Determination of GAA metabolites in small intestinal mucosal tissue

The frozen intestinal mucosal tissues were ground along with liquid nitrogen. To 0.8 g of the ground tissues, 1 mL of distilled water was added. The mixture was then vortexed for 3 min, subjected to ultrasonic extraction for 10 min, and centrifuged at 12,000 rpm for 10 min. Then, 200 μL of the supernatant was transferred to another centrifuge tube. To this, 400 μL of acetonitrile was added, mixed well, and allowed to stand for 10 min to precipitate proteins. The sample was then centrifuged at 12,000 rpm for 10 min, and 100 μL of the supernatant was mixed with 200 μL of phosphoric acid (2 mM). From this sample, 20 μL was used for high‐performance liquid chromatographic analysis. Determination conditions: IC YS‐50 weak acid cation exchange column (4.6 mm × 125 mm) was used; the flow rate was 1.0 mL/min; the column temperature was 30°C; the detection wavelength was 210 nm; the elution mode was one‐time linear elution; the sample size was 20 μL.

#### 2.5.4 Conventional nutrient determination

Dry matter (DM) content in the diet and faecal samples was determined according to GB/T 6435‐2014, organic matter (OM) according to GB/T 6438‐2007 and ether extract (EE) according to GB/T 6433‐2006. In addition, neutral detergent fibre (NDF) and acid detergent fibre (ADF) were determined using Van's detergent fibre analysis, crude protein (CP) using the Kjeldahl method, calcium using the *o*‐cresolphthalein colorimetric method and phosphorus using the ammonium vanadium‐molybdate colorimetric method.

#### 2.5.5 Transcriptome analysis and real‐time polymerase chain reaction (PCR) of intestinal mucosa

Four sheep of test groups I and II, which were close to the group average BW, were selected to do the transcriptome analysis. Frozen mucosal samples of the duodenum and jejunum were sterilized and then ground with liquid nitrogen. Subsequently, Trizol reagent was used to extract the total RNA from the samples. In total, 16 RNA samples were obtained from duodenal and jejunal mucosal tissues. RNA sequencing was performed using the Agilent 2100 bioanalyzer to precisely determine RNA concentration, total amount and integrity, as reported previously (Fu et al., 2023; Zhao et al., 2023). In addition, RNA samples were tested for constructing an intestinal transcriptome library and transcriptome sequencing.

Primer sequences and internal reference genes were determined using the Oligo 7.0 software Table S1). In total, six genes that were significantly differentially expressed in duodenal mucosal tissue (SI and LOC101109677) and in jejunal mucosal tissue (GCK, ACACA, FASN and DHFR) were validated using qRT‐polymerase chain reaction. The experiment was repeated at least thrice for all samples, using GAPDH as the internal reference.

### 2.6 Statistical and bioinformatics analysis

The raw data obtained were organized using Microsoft Excel 2013. The data on growth performance, nutrient digestibility and intestinal mucosal and plasma measurements of each group were analysed and processed using one‐way analysis of variance. All data were statistically analysed using SAS 9.2, and the significant differences were assessed multiple times via the Duncan's method. Data are expressed as means and standard errors of the mean. Results with *p* < 0.05 are significant, with *p* < 0.01 are highly significant and with 0.05 ≤ *p* ≤ 0.10 exhibit a trend of statistical difference.

The raw sequencing data from the current study have been deposited in the NCBI database with the accession number PRJNA1076840. The imaging data of sequenced fragments obtained using the high‐throughput sequencer were converted into sequence data via CASAVA base identification, and the raw data were filtered by removing reads with connectors (adapter), removing reads containing N (base information; cannot be determined) and removing low‐quality reads (reads with the number of bases with Qphred ≤20 accounting for >50% of the entire read length). In addition, Q20, Q30 and GC contents were calculated using clean data. All subsequent high‐quality analyses were performed using clean data. HISAT2 v2.0.5 was used to construct the reference genome index as well as to compare paired end‐clean reads with the reference genome. The number of reads mapped to each gene was determined. Subsequently, the expected number of transcript sequence fragments per kilobase of transcript per million mapped was calculated for each gene based on the gene length. In addition, the number of reads mapped to that gene was calculated. Gene ontology (GO) enrichment analysis of DEGs was performed using clusterProfiler (3.4.4), and GO terms with corrected *p*‐values <0.05 were considered significantly enriched by DEGs. Statistical enrichment of DEGs in the KEGG pathway was analysed using the clusterProfiler (3.4.4) software. Based on the transcriptome gene analysis, six genes were selected for real‐time ‐qPCR to validate the transcriptome model and result accuracy.

## 3 RESULTS

### 3.1 Effect of GAA and RPB supplementation on the BW, ADG and DMI of sheep

As shown in Table 2, compared with control group and test group I, the ADG in test group II increased by 12.88% (*p* < 0.01) and 8.76% (*p* < 0.05), respectively, during the experimental period from 1 to 30 days. On the contrary, the FCR of test group II was lower (*p* < 0.05) than that of control group and test group I. There were no significant differences in DMI (*p* > 0.05) among three groups. Besides, GAA and RPB supplemented during 31–60 days and 1–60 days revealed no significant differences in feed intake, ADG and mean FCR (*p* > 0.05).

**TABLE 2:** Effects of dietary GAA and rumen‐protected betaine (RPB) on the BW, ADG and DMI of sheep.

Items	Control group (*n* = 6)	Test group I (*n* = 6)	Test group II (*n* = 6)	SE	*p*‐Value
Initial BW, kg	27.47	27.45	27.45	0.32	1.00
30‐day BW, kg	34.07	34.30	34.90	0.37	0.66
60‐day BW, kg	39.50	39.77	40.17	0.34	0.75
1–30 days					
DMI, g/day	1271.38	1296.00	1267.77	9.70	0.46
ADG, g/day	220.00^bB^	228.33^bAB^	248.33^aA^	4.49	0.02
FCR	5.78^a^	5.67^a^	5.10^b^	0.13	0.03
31–60 days					
DMI, g/day	1440.74	1444.67	1477.76	10.05	0.27
ADG, g/day	181.11	182.22	175.63	4.85	0.85
FCR	7.95	7.93	8.41	0.25	0.60
1–60 days					
DMI, g/day	1356.10	1370.34	1372.77	9.09	0.74
ADG, g/day	200.56	205.28	211.94	2.68	0.23
FCR	6.76	6.67	6.48	0.10	0.43

*Note*: Control group, fed basal diet; test group I, fed basal diet and supplemented with 1500 mg/kg GAA; test group II, fed basal diet and supplemented with 1500 mg/kg GAA + 600 mg/kg RPB. The data in the same row marked with different lowercase letters are significantly different (*p* < 0.05), and different uppercase letters indicate highly significant differences (*p* < 0.01), whereas same letters or without letters indicate no significant differences (*p* > 0.05).

Abbreviations: ADG, average daily gain; BW, body weight; DMI, dry matter intake; FCR, feed conversion ratio; GAA, guanidine acetic acid; SE, standard error.

### 3.2 Effect of GAA and RPB supplementation on apparent digestibility of nutrients in diets

Table 3 shows the apparent digestibility of nutrients in diets at 30 and 58 days. No obvious difference of DM, OM, CP, NDF, ADF and calcium apparent digestibility was found among all groups on day 30 (*p* > 0.05). Nevertheless, compared with control group and test group I, the EE apparent digestibility of test group II was increased by 6.76% and 4.11% (*p* < 0.01), respectively. In addition, the apparent digestibility of phosphorus in test group II was higher than that of test group I (*p* < 0.05) and control group (*p* < 0.01). On day 58, the apparent digestibility of DM, OM, CP, NDF, ADF, calcium and phosphorus did not differ significantly among the groups (*p* > 0.05). However, the EE apparent digestibility of test group II was increased by 7.75% and 5.79% when compared to control group and test group I (*p* < 0.01).

**TABLE 3:** Effects of GAA and rumen‐protected betaine (RPB) supplementation on apparent digestibility of nutrients in diets on days 30 and 58 (%).

Items		Control group (*n* = 6)	Test group I (*n* = 6)	Test group II (*n* = 6)	SE	*p*‐Value
DM	day 30	63.59	65.77	66.08	0.57	0.15
	day 58	62.23	64.16	63.94	0.79	0.58
OM	day 30	63.69	65.82	66.84	0.62	0.10
	day 58	60.41	62.21	62.04	0.84	0.66
CP	day 30	62.27	65.09	65.96	1.01	0.31
	day 58	65.30	65.03	65.24	1.10	0.99
EE	day 30	71.26^B^	73.08^B^	76.08^A^	0.60	<0.01
	day 58	71.78^B^	73.11^B^	77.34^A^	0.79	<0.01
NDF	day 30	46.43	49.94	49.40	0.90	0.24
	day 58	47.23	49.65	51.41	1.12	0.33
ADF	day 30	32.34	36.55	35.77	0.88	0.11
	day 58	35.33	36.06	37.45	1.34	0.83
Calcium	day 30	31.41	33.32	33.96	0.97	0.56
	day 58	32.29	32.75	31.36	1.35	0.92
Phosphorus	day 30	28.04^bB^	29.72^bAB^	34.54^aA^	0.98	0.01
	day 58	29.02	29.27	32.70	1.00	0.26

*Note*: Control group, fed basal diet; test group I, fed basal diet and supplemented with 1500 mg/kg GAA; test group II, fed basal diet and supplemented with 1500 mg/kg GAA + 600 mg/kg RPB. The data in the same row marked with different lowercase letters are significantly different (*p* < 0.05), and different uppercase letters indicate highly significant differences (*p* < 0.01), whereas same letters or without letters indicate no significant differences (*p* > 0.05).

Abbreviations: ADF, acid detergent fibre; CP, crude protein; DM, dry matter; EE, ether extract; GAA, guanidine acetic acid; NDF, neutral detergent fibre; OM, organic matter; SE, standard error.

### 3.3 Effect of GAA and RPB supplementation on plasma parameters in sheep

At day 30, the creatine level in plasma obtained from the jugular vein was increased by 12.62% and 9.11% in test group II as compared with control group and test group I (*p* < 0.05) (Table 4). However, GAA and creatinine levels were not different among the groups (*p* > 0.05). At day 60, plasma GAA levels were 7.74% (*p* < 0.05) and 10.58% (*p* < 0.01) higher in test group II than those in test group I and the control group, respectively. Moreover, the creatine and creatinine levels were similar among the groups (*p* > 0.05).

**TABLE 4:** Effects of GAA and betaine supplementation on plasma parameters of sheep.

Items	Control group (*n* = 6)	Test group I (*n* = 6)	Test group II (*n* = 6)	SE	*p*‐Value
30 days					
GAA, μmol/L	196.18	203.58	201.22	4.43	0.81
Creatine, μmol/L	116.44^b^	120.19^b^	131.14^a^	2.46	0.03
Creatinine, μmol/L	57.50	56.37	54.46	1.43	0.71
60 days					
GAA, μmol/L	206.85^bB^	212.31^bAB^	228.74^aA^	3.53	0.02
Creatine, μmol/L	158.76	162.64	166.32	3.08	0.63
Creatinine, μmol/L	42.66	39.78	37.99	1.58	0.71

*Note*: Control group, fed basal diet; test group I, fed basal diet and supplemented with 1500 mg/kg GAA; test group II, fed basal diet and supplemented with 1500 mg/kg GAA + 600 mg/kg RPB. The data in the same row marked with different lowercase letters are significantly different (*p* < 0.05), and different uppercase letters indicate highly significant differences (*p* < 0.01), whereas same letters or without letters indicate no significant differences (*p* > 0.05).

Abbreviations: GAA, guanidine acetic acid; SE, standard error.

### 3.4 Effect of GAA and RPB supplementation on plasma GAA levels in the small intestinal mucosa and superior mesenteric vein of sheep

Compared with the control group and test group I, test group II had higher GAA content in the duodenal mucosal tissue (*p* < 0.01). In addition, the GAA content in the jejunal mucosal tissue was higher (*p* < 0.01) than that in test group I. The GAA content in the ileac mucosal tissue and the superior mesenteric vein of test group II was higher (*p* < 0.01) than those in the control group and the control group (*p* < 0.01) (Table 5).

**TABLE 5:** Effects of GAA and rumen‐protected betaine (RPB) supplementation on GAA and creatine contents in the small intestine of sheep.

Items		Control group (*n* = 6)	Test group I (*n* = 6)	Test group II (*n* = 6)	SE	*p*‐Value
Tissue						
Duodenum, μg/g	GAA	373.71^B^	382.09^B^	442.14^A^	10.30	<0.01
	creatine	373.19^bB^	403.76^bAB^	453.48^aA^	10.33	<0.01
Jejunum, μg/g	GAA	461.89^bAB^	433.49^bB^	518.40^aA^	11.92	<0.01
	creatine	373.19^bB^	403.76^bAB^	453.48^aA^	10.33	<0.01
Ileum, μg/g	GAA	278.10^bB^	292.01^bAB^	334.63^aA^	8.61	<0.01
	creatine	584.64^a^	522.55^b^	557.74^ab^	11.66	0.03
Plasma						
Superior mesenteric vein, μmol/L	GAA	237.02^bB^	253.36^bAB^	280.09^aA^	6.09	<0.01
	creatine	138.34^b^	148.93^ab^	163.02^a^	4.37	0.02

*Note*: Control group, fed basal diet; test group I, fed basal diet and supplemented with 1500 mg/kg GAA; test group II, fed basal diet and supplemented with 1500 mg/kg GAA + 600 mg/kg RPB. The data in the same row marked with different lowercase letters are significantly different (*p* < 0.05), and different uppercase letters indicate highly significant differences (*p* < 0.01), whereas same letters or without letters indicate no significant differences (*p* > 0.05).

Abbreviations: GAA, guanidine acetic acid; SE, standard error.

### 3.5 Effect of GAA and RPB supplementation on plasma creatine levels in the small intestinal mucosa and superior mesenteric vein of sheep

The creatine content in the sheep duodenal mucosa was higher (*p* < 0.05) in test group II than that in the control group and test group I. However, the creatine content in the ileal mucosa in test group II was not significantly different from that in the other groups (*p* > 0.05); it was, however, significantly lower in test group I than in the control group (*p* < 0.05). In addition, the creatine content in the jejunal mucosa was not different among the groups (*p* > 0.05). The creatine content in the plasma obtained from the superior mesenteric vein was higher in test group II than in the control group (*p* < 0.05) (Table 5).

### 3.6 Effects of GAA and RPB supplementation on the small intestinal mucosa of sheep determined via transcriptome analysis

#### 3.6.1 Quantification and testing of RNA extracted from the intestinal mucosa

Four mucosal samples each from the duodenum and jejunum were selected for RNA extraction each from test groups I and II. As shown in Table S2, according to the 260/280 nm ratio (1.8–2.1), RNA concentration and RNA integrity number to identify the detection grade, the grade selected high‐quality RNA samples for library construction, respectively. The total RNA in each intestinal mucosal sample was >31 μg, and the mean RNA integrity in the duodenal mucosa was 7.33 and 8.13 in test groups I and II, respectively. Moreover, the mean RNA integrity of the jejunal mucosa was 8.23 and 7.65 in test groups I and II, respectively, indicating high RNA integrity. The results showed that the intestinal mucosa used in the study successfully met the quality standards and satisfied the conditions of the subsequent tests for further analysis.

#### 3.6.2 Quality of intestinal mucosa sequencing data

The summary statistics of sequence quality and alignment information for the 16 intestinal mucosal samples are shown in Table S3. The raw reads in the duodenal and jejunal mucosa of test groups I and II were de‐streeted, de‐N‐base and mass shear filtered, and the number of bases obtained from the 16 samples after filtration was above 6G. In addition, the rate of sequencing error of this test was not >0.03%, and the percentage of bases G and C in the filtered reads was 50.8%–53.39%. The quality values of bases in all reads were >30, and usually, the number of bases with base quality values (Phred) above 20 (Q20) was >85% to meet the sequencing requirements. Moreover, the Q20 in this test was >96.74%, and the Q30 was >90.85%. The results showed that the samples were free of contamination, the sequencing error rate was low, and the sequencing quality was high. In addition, the statistical results of the data from 16 samples all met the basic requirements of transcriptome sequencing, and the data met the conditions of subsequent analysis.

#### 3.6.3 Comparison of the intestinal mucosa with the sheep reference genome

The results of the contrast ratio analysis of intestinal mucosal samples are shown in Table S4. The 16 intestinal mucosal samples were compared with sheep reference genome Oar_v3.1, and the proportion of filtered reads in exons ranged from 80.99% to 89.41% and in introns ranged from 5.5% to 13.39%, indicating that the filtered reads were obtained by sequencing. The total mapped ratio was >92.86%, the unique mapped ratio was >82.51%, and the multiple mapped ratio (percentage of reads that matched with multiple positions in the reference genome) ranged from 7.07% to 11.97%. The results showed that the proportion of the generated sequencing reads had >80% similarity with the reference genome, indicating that the selected reference genome met the sequencing conditions and that the sequencing data were reliable.

#### 3.6.4 Analysis of DEGs

**FIGURE 1:**
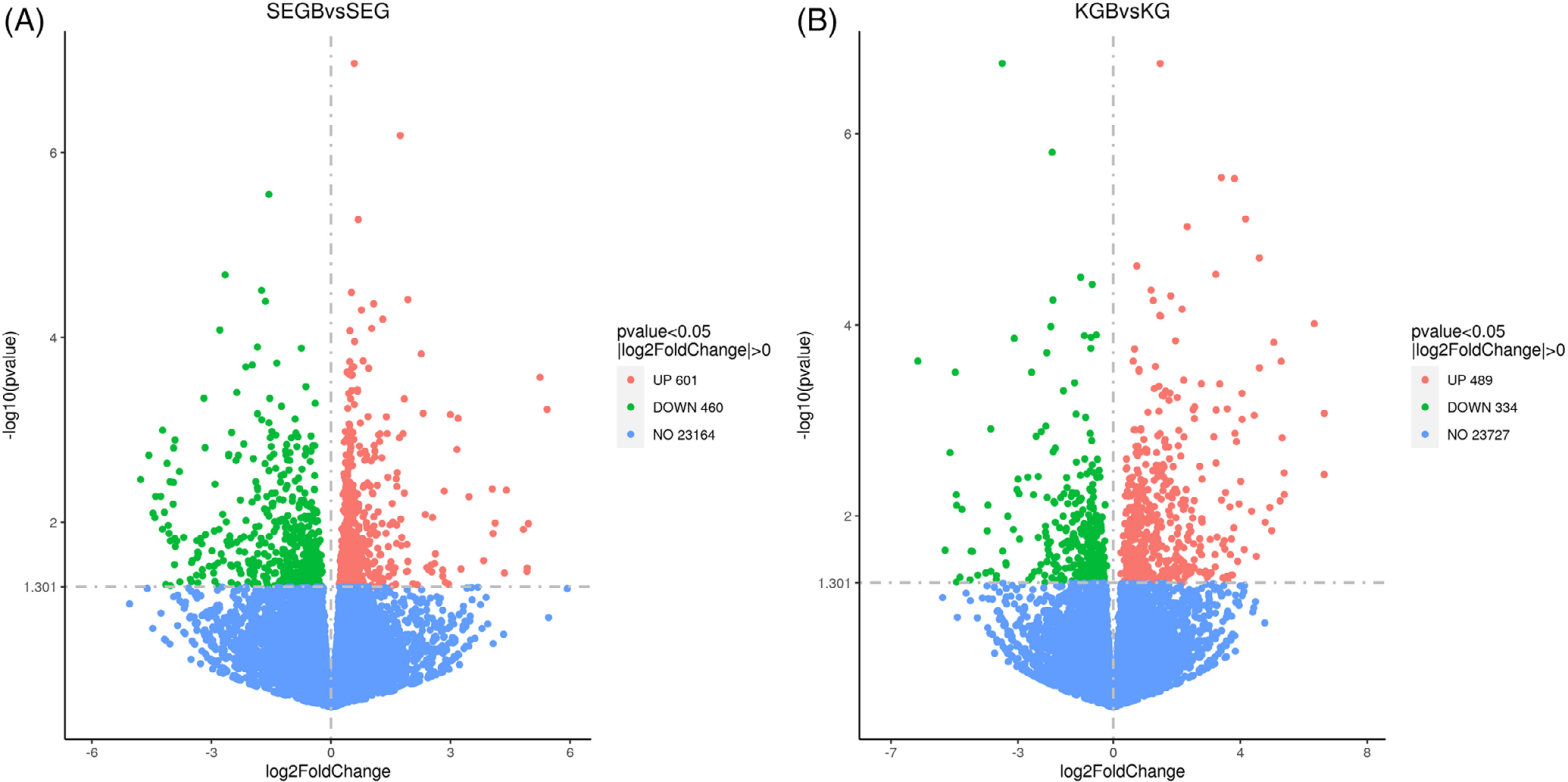
The volcano plot of differentially expressed genes in the duodenal (A) and jejunal (B) mucosa. SEG is duodenal mucosa of test group I, SEGB is duodenal mucosa of test group II, KG is jejunal mucosa of test group I, KGB is jejunum mucosa of test group II. Red dots represent upregulated genes, green dots represent downregulated genes, and blue dots represent genes with no significant differences.

According to the volcano map of DEGs in the intestinal mucosa (Figure 1), the total number of genes that correlated with the duodenal genome was 24,225 (Figure 1A) and with the jejunal genome was 24,550 (Figure 1B). Between test groups II and I, DEGs were identified at significant levels of *p* < 0.05 and | log2FoldChange | >0. The number of DEGs noted in the duodenum and jejunum was 1061 and 823, respectively. Among them, 601 genes were upregulated and 460 genes were downregulated in the duodenal mucosa tissue, and 489 genes were upregulated and 334 genes were downregulated in the jejunal mucosal tissue.

**FIGURE 2:**
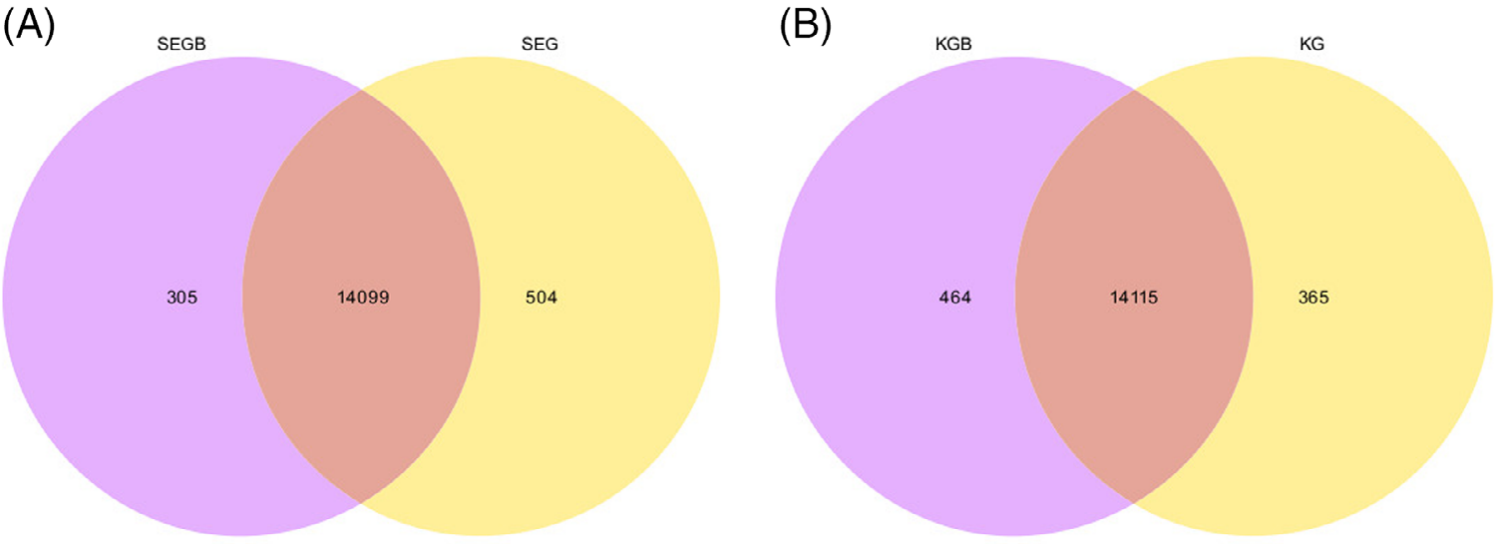
Venn diagram of differentially expressed genes in the duodenal (A) and jejunal (B) mucosa. SEG is duodenal mucosa of test group I, SEGB is duodenal mucosa of test group II, KG is jejunal mucosa of test group I, KGB is jejunum mucosa of test group II.

Figure 2 shows the Venn diagram of DEGs in the intestinal mucosal tissues. In total, 14,908 genes were identified in the duodenal mucosal tissues, of which 14,099 were shared between test groups I and II, 504 were specifically expressed in test group I, and 305 were specifically expressed in test group II (Figure 2A). In total, 14,944 genes were observed in jejunal mucosal tissues, of which 14,115 were shared between test groups I and II, 365 were specifically expressed in test group I, and 464 were specifically expressed in test group II. The total number of genes in the jejunal mucosal tissue was 14,944, among which 14,115 were shared between test groups I and II, 365 were specifically expressed in test group I, and 464 were specifically expressed in test group II (Figure 2B).

#### 3.6.5 GO enrichment analysis of DEGs in the small intestine

**FIGURE 3:**
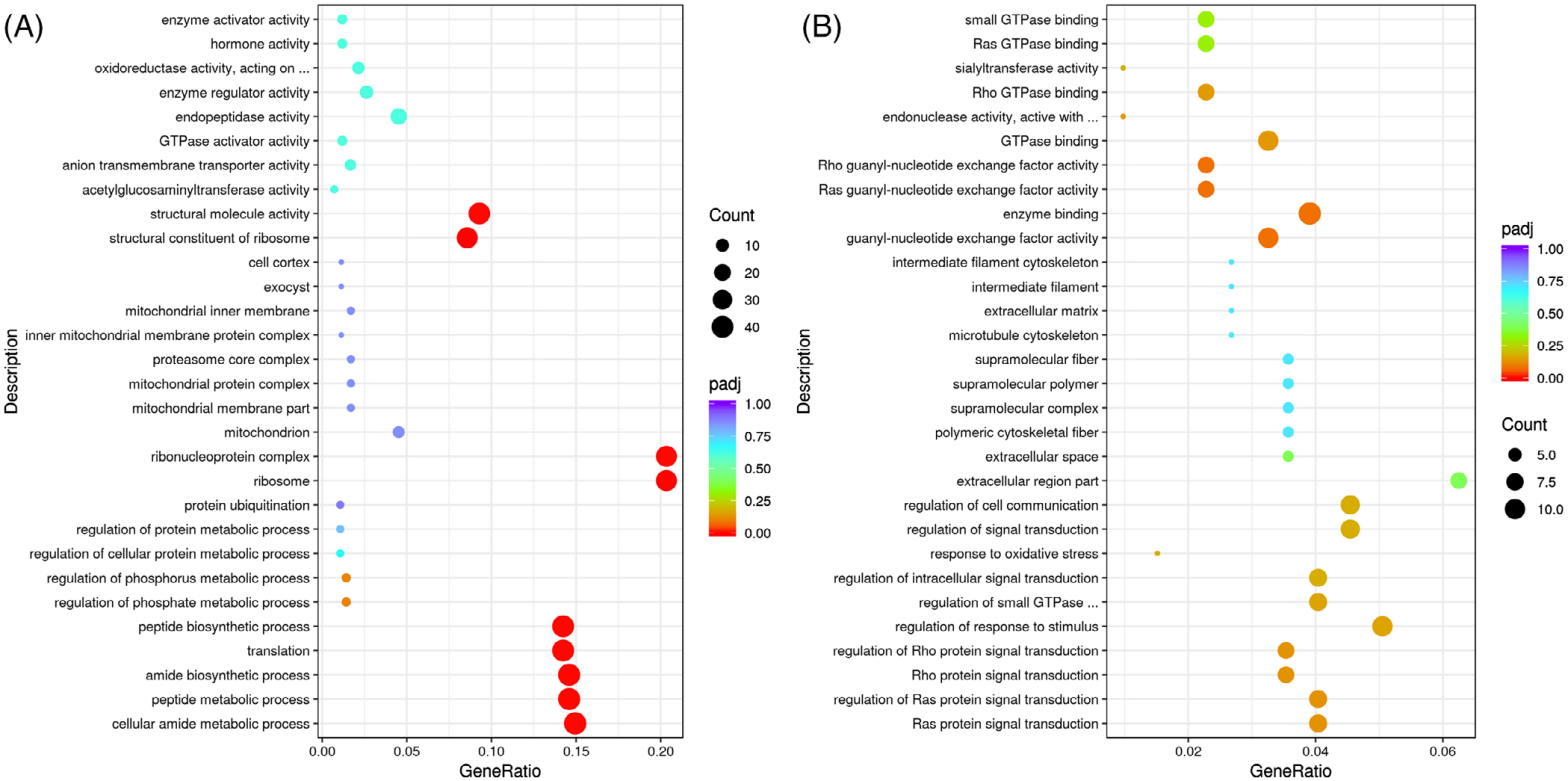
Gene ontology (GO) analysis of differentially expressed genes in the duodenal (A) and jejunal (B) mucosa.

In the test groups, DGEs were screened from different intestinal segments and were subjected to GO analysis (Figure 3). The GO database classifies genes into three domains according to their functions: biological process (BP), cellular component (CC) and molecular function (MF). Figure 3A shows the GO terms significantly associated with the DEGs in the duodenal mucosa. The top 10 most significantly enriched GO terms related to DEGs were cellular amide metabolism (BP), peptide metabolism (BP), amide biosynthetic process (BP), translation (BP), peptide biosynthesis (BP), phosphate metabolic regulation (BP), ribosome (CC), ribonucleoprotein complex (CC), structural constituent of ribosome (MF) and structural molecule activity (MF).

As shown in Figure 3B, in jejunal mucosa, the top 10 significantly enriched GO terms related to DEGs were guanyl‐nucleotide exchange factor activity (MF), enzyme binding (MF), Ras guanyl‐nucleotide exchange factor activity (MF), Rho guanyl‐nucleotide exchange factor activity (MF), Ras protein signal transduction (BP), Ras protein signal transduction regulation (BP), Rho protein signal transduction (BP), Rho protein signal transduction regulation (BP), extracellular region part (CC) and extracellular space (CC).

#### 3.6.6 KEGG pathway enrichment analysis of small intestine segments with different genes

**FIGURE 4:**
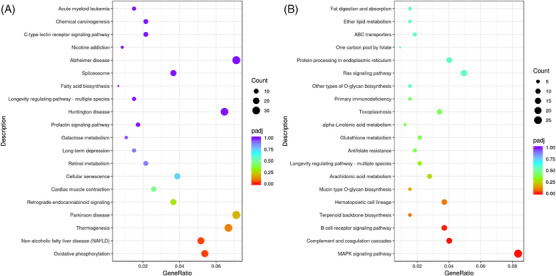
KEGG analysis of differentially expressed genes in the duodenal (A) and jejunal (B) mucosa.

As shown in Figure 4A, the top 20 KEGG pathways associated with the DEGs in the duodenal mucosa of test groups II and I were as follows: oxidative phosphorylation, non‐alcoholic fatty liver disease, thermogenesis, retrograde endocannabinoid signalling, cardiac muscle contraction, cellular senescence, retinol metabolism, galactose metabolism, prolactin signalling pathway, Huntington disease, longevity regulating pathway—multiple species, fatty acid biosynthesis, spliceosome, Alzheimer's disease, nicotine addiction, C‐type lectin receptor signalling pathway, chemical carcinogenesis and acute myeloid leukaemia.

As shown in Figure 4B, the top 20 KEGG pathways associated with DEGs in the jejunal mucosa of test groups II and I were as follows: MAPK signalling pathway, complement and coagulation cascades, B‐cell receptor signalling pathway, terpenoid backbone biosynthesis, hematopoietic cell lineage, mucin type *O*‐glycan biosynthesis, arachidonic acid metabolism, longevity regulating pathway—multiple species, antifolate resistance, glutathione metabolism, alpha‐linolenic acid metabolism, toxoplasmosis, primary immunodeficiency, other types of *O*‐glycan biosynthesis, Ras signalling pathway, protein processing in endoplasmic reticulum, one carbon pool by folate, ABC transporters, ether lipid metabolism and fat digestion and absorption.

#### 3.6.7 Validation of DEGs using transcriptome analysis

**FIGURE 5:**
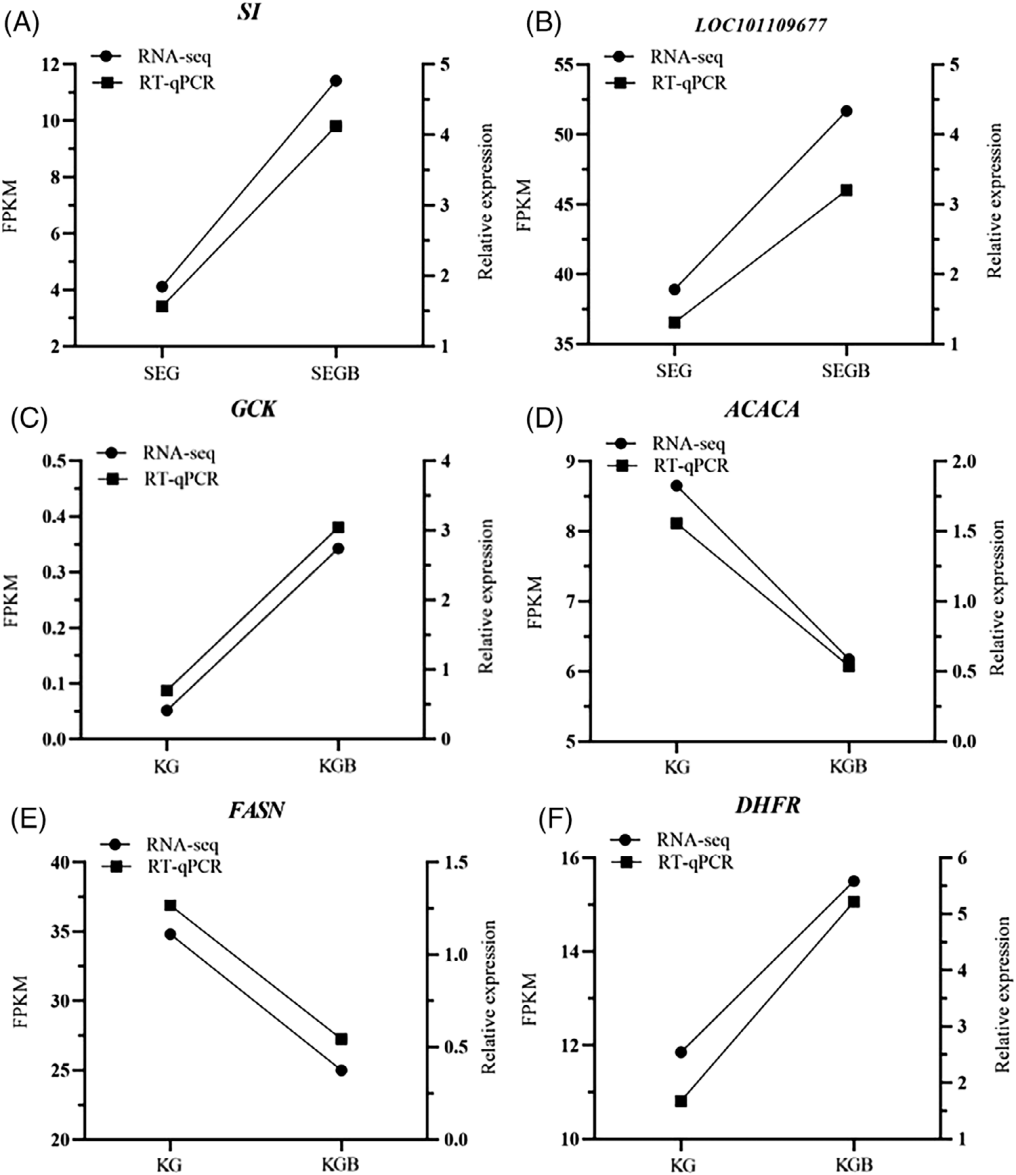
Comparison of trends in real‐time (RT)‐qPCR data and RNA‐seq data for differently expressed genes. SI is sucrase‐isomaltase gene, LOC101109677 is intestinal‐type alkaline phosphatase gene, GCK is glucokinase gene, ACACA is acetyl‐CoA carboxylase alpha gene, FASN is fatty acid synthase gene, DHFR is dihydrofolate reductase gene. SEG is duodenal mucosa of test group I, SEGB is duodenal mucosa of test group II, KG is jejunal mucosa of test group I, KGB is jejunum mucosa of test group II.

Of all DEGs, the relative expressions of SI and LOC101109677 in the duodenum, of GCK and DHFR and of ACACA and FASN in the jejunum were higher in test group II than in test group I (Figure 5). The trend was consistent with the results of transcriptomic RNA‐seq, indicating a high degree of confidence in the results of transcriptome analysis.

## 4 DISCUSSION

Creatine stores energy through high‐energy phosphate bonds, owing to which it serves as an energy source for muscles. In the kidneys, creatine is synthesized via GAA as the only precursor; moreover, creatine is synthesized endogenously in the liver via GAA methylation, which highlights the crucial role of GAA. Particularly, in livestock and poultry, most studies have clarified that the growth phase is associated with a high demand for creatine. As a result, the supplementation of creatine and GAA in diets can increase growth performance and improve feed utilization in cattle (Ardalan et al., 2020), broiler (Sciebce, 2012) and pigs (Wang et al., 2012). Structurally, in GAA, SAM provides the methyl groups required for creatine synthesis. Thus, 40%–75% of the methyl groups in SAM involved in the methionine cycle are involved in the GAA‐mediated synthesis of creatine (Edison et al., 2007). Reportedly, betaine provides methyl groups sufficient for meeting the body's creatine requirement, when supplied along with GAA. In addition, betaine is involved in amino acid metabolism and fat metabolism, which can promote animal feeding and growth, increase feed conversion efficiency, and improve meat quality (Hwangbo et al., 2015; Rojas‐Cano et al., 2016). During the experimental period of 1–30 days in the current study, supplementation with 1500 mg/kg GAA and 600 mg/kg RPB significantly increased the ADG of sheep, indicating that the combined supplementation can improve the early growth performance of sheep.

However, during the whole experimental period, the growth performance did not show significant difference among all treatments. Unfortunately, the dietary energy level of the current experiment was lower than the energy required for sheep gain weight at 200 g/day. This may be one reason that adding GAA and RPB had no effect on ADG of sheep at the later period of the feeding trial. According to the results, we speculated that GAA and RPB supplementation can promote growth by improving glucolipid metabolism. The potential mechanism of action needs further exploration. In general, the Kazakh sheep are usually grazed in summer. Thus, we designed a relatively lower energy level in the diet. In the future, we will do more experiment in sheep including reduction of adding time of GAA and RPB and comparison of different energy levels of diet.

In monogastric animal studies, creatine has been shown to play crucial roles in digestive fluid secretion, nutrient absorption, renewal of digestive tract cells and phosphocreatine supply system in cells (Sistermans et al., 1995). As a result, supplementation with GAA has been shown to improve intestinal morphology (Amiri et al., 2019). In ruminants, GAA supplementation increases the apparent digestibility of DMI, OM, CP, NDF and ADF (Liu & Chen et al., 2021; Li et al., 2020). In addition, betaine supplementation is associated with increased apparent digestibility of DMI, OM and CP in fattened cattle as well as significantly increased apparent digestibility of nutrients in goats (Wafaa & Reham, 2023; Wang et al., 2020). In the present study, we found that GAA and RPB supplementation improved the apparent digestibility of EE and phosphorus in the first and second parts of the experiment, respectively. Our results indicated that sheep could obtain more energy through enhanced EE absorption caused by GAA and RPB supplementation, which was conducive to improving growth performance of sheep. A study by Liu & Wang et al. (2021) demonstrated that in Angus bulls, dietary supplementation with 0.6 g/kg GAA and 0.6 g/kg betaine significantly increased the 60‐day BW, ADG and feed efficiency as well as significantly increased the apparent digestibility of DMI, OM, CP and ADF. In addition, the supplementation significantly increased the rumen levels of *Flavobacterium* and *Fibrobacter succinogenes* and plasma levels of creatine (Liu et al., 2021). Thus, GAA and betaine can promote the absorption and metabolism of nutrients in the digestive tract. The positive effects may be related to the fact that GAA and RPB directly improve nutrient digestion by promoting the activity of digestive enzymes and enrich intestinal microbiota to maintain the intestinal microecological balance through osmotic pressure regulation. In addition, GAA and betaine promote fat digestion and absorption, mainly due to the role of betaine in promoting lipolysis. Specifically, betaine regulates lipid metabolism by providing active methyl groups, which promote phospholipid synthesis in vivo, as well as regulating lipid synthase and carnitine activity (Sang et al., 2012).

Creatine plays an important role in regulating energy metabolism within cells. Reportedly, creatine is sourced via two routes: 50% from endogenous synthesis and 50% from ration supply. The endogenous synthesis of creatine mainly involves three amino acids: arginine, glycine and methionine, and 85% of the GAA methylation reaction to form creatine occurs in the liver. Exogenous supplementation of GAA can prevent the use of these amino acids in the synthesis of creatine by instead providing methyl via the methionine cycle. Reportedly, in lactating piglets, supplementation of GAA and methionine significantly increased creatine levels in the kidneys and liver than supplementation with GAA alone, which suggested that more methionine was required to be metabolized to convert GAA to creatine to provide methyl (Chandani et al., 2021). In our experiment, plasma GAA and creatine levels increased with time, indicating that the exogenous supplementation of GAA and RPB can promote GAA and creatine synthesis in sheep, which improves the organism's energy metabolism. In addition, creatine synthesis was higher with exogenous supplementation of both GAA and RPB than with supplementation of GAA alone, indicating that adding betaine enhanced methyl supply to facilitate increased creatine synthesis in sheep.

The liver is the main tissue for creatine synthesis, and other organs that can synthesize creatine include the pancreas, kidneys, brain and testis. The brain and testis creatine production may not depend on the liver and pancreas (Braissant et al., 2001; Moore, 2001; Ringel et al., 2008). However, Dinesh et al. (2019) demonstrated that the intestine of newborn piglets can synthesize GAA and creatine in large amounts, and the net release of GAA and creatine from the intestinal weave increased with the supplementation of arginine + methionine and creatine + arginine + methionine. In addition, l‐arginine glycine methyltransferase and guanidinoacetate amidino transferase were detected but had low activity. In this study, supplementary feeding of GAA and betaine increased GAA levels in the mucosal tissue of each intestinal segment and the plasma obtained from the superior mesenteric vein and duodenal mucosa. The superior mesenteric vein collects venous blood from the duodenum up to the intestine above the left flexure of the colon, part of the stomach, and pancreas. This finding is consistent with the above study, suggesting that the supplementation of GAA and betaine in feed can affect the synthesis of GAA and creatine in small intestinal segments. We additionally found that GAA and betaine supplementation increased the substrate availability for creatine synthesis in the sheep intestine, reducing endogenous protein catabolism and increasing creatine anabolism, which increased the content of GAA and creatine in the intestinal mucosa (Dinesh et al., 2019).

In transcriptomic assays, we found that the addition of RPB to GAA could promote the expression of specific genes in the epithelial cells of the sheep small intestine. The duodenal GO analysis revealed that the DEGs related to the BPs of cellular amide metabolic processes, peptide metabolic processes, amide biosynthetic processes, translation, peptide biosynthetic processes and phosphate metabolic process regulation were significantly enriched. These findings suggested that RPB and GAA supplementation could regulate the protein metabolism, which had positive influence on healthy growth of sheep. In the jejunum, DEGs associated with the MAPK signalling pathway, complement and coagulation cascade and B‐cell receptor signalling pathway were significantly enriched. These pathways are mainly involved in energy metabolism, methyl metabolism and fatty acid β‐oxidation involved in small intestinal ATP, indicating that GAA and RPB could improve energy absorption of sheep, which was in accordance with result of EE digestibility.

We additionally found that DEGs involved in oxidative phosphorylation, cAMP signalling pathway and AMPK signalling pathway of energy metabolism were upregulated in the duodenum. In the jejunum, DEGs associated with the AMPK signalling pathway, ABC transport system, cAMP signalling pathway and oxidative phosphorylation were upregulated. In the presents study, the duodenal KEGG pathways of arginine biosynthesis, folate biosynthesis, cysteine and methionine metabolism and the jejunal KEGG pathways of anti‐folic acid, one‐carbon pool folate and folate biosynthesis were associated with creatine metabolism or methyl metabolism. The gastrointestinal tract and liver are jointly responsible for the release of amino acids into the peripheral blood, which in turn supports protein synthesis. The intestine is an active site for amino acid metabolic reactions; moreover, the metabolism of many essential and non‐essential amino acids produces products that are important for overall metabolic function. For example, essential amino acids such as methionine are involved in protein synthesis, methyl donor supply and SAM formation, and non‐essential amino acids or amino acid derivatives, such as arginine, betaine, choline and taurine (Wu, 2009). Arginine plays an important role in regulating nutrient metabolism, growth and development of animals by participating in functions, such as protein synthesis, cell proliferation, DNA synthesis and cell protection and migration (Corl et al., 2008; Tan et al., 2010; Tan et al., 2015). Transcriptome analysis has revealed that duodenal arginine biosynthesis is downregulated by two genes: nitric oxide synthase 2 (which inhibits the generation of arginine succinate from aspartate) and argininosuccinate synthase 1 (which inhibits the interconversion of arginine with citrulline). Notably, arginine and glycine can synthesize GAA; however, as supplementation of betaine and GAA increases GAA levels, decreasing the rate of arginine synthesis can save arginine. The addition of betaine to the jejunal anti‐folate acid, one‐carbon pool folate and folate biosynthetic pathways promotes high expression of DHFR, an oxidoreductase that uses NADPH to reduce dihydrofolate to produce tetrahydrofolate.

The solute carrier (SLC) superfamily mediates the transport of various solutes, including sugars, amino acids, peptides and peptide‐like drugs, across membranes between cells and inside/outside the cell (Claire et al., 2016; Liu, 2019; Zhang et al., 2019). In the present study, several SLC family genes were significantly expressed in the duodenum, including SLC25A6, an adenine nucleotide transporter protein for reverse ATP/ADP transport, SLC26A3 for bicarbonate transport, SLC52A2 for riboflavin transporter protein, SLC35B1 for galactose transporter protein, SLC6A14 for basic amino acid transport with Na^+^ dependency, SLC16A1 for monocarboxylic acid transport, SLC13A4 for sodium/sulphate cotransport, SLC9A3R2 for protein binding and SLC7A10 for neutral amino acid transport with Na^+^ dependence. In jejunal mucosa, the following genes were significantly upregulated: SLC19A2, responsible for transporting folic acid and vitamin B1; SLC13A1, a citric acid cycle intermediate transporter responsible for sodium/sulphate cotransport; SLC5A5, a sodium iodide co‐transport protein involved in Na^+^ transport; SLC52A3, responsible for transporting riboflavin; and SLC16A4, a monocarboxylate transporter protein. The results of the above analysis showed that most of the transporter proteins were significantly or highly significantly upregulated. This additionally indicated that most transporter substrates, including sugars, amino acids, inorganic salt ions, vitamins, peptides and other small molecules, were transported in the intestine, which facilitated the digestion and absorption of nutrients in the intestine.

Sucrase, maltase and aminopeptidase in the brush border membrane of the small intestine can initially respond to the absorption of sugars and proteins by the organism (Matthias & Tobias, 2005). Alkaline phosphatase is a phosphate monoester hydrolase, an active enzyme that is excreted by the liver via bile and that plays an important role in the mucosal mechanical (Lisle et al., 2011), chemical (Mizumori et al., 2009), biological (Jean‐Paul, 2010) and immune barriers (Bates et al., 2007). In this study, several genes in the duodenal and jejunal mucosa were associated with glucose metabolic processes, such as galactose metabolism, carbohydrate digestion and absorption, insulin signalling pathway, mTOR signalling pathway, insulin secretion and glucagon signalling pathway. Among them, galactokinase 1 (GALK1), lactase (LCT) and maltase (SI) were significantly upregulated in the galactose metabolic pathway. GALK1 can promote the conversion of α‐d‐galactose to α‐d‐galactose‐1P, LCT can regulate lactose to α‐d‐glucose, and SI can convert sucrose to d‐fructose. In addition, LCT and SI are involved in the carbohydrate digestion and absorption pathway. This study found that GAA combined with betaine can promote intestinal creatine synthesis, and reportedly, creatine, betaine and choline can maintain normal intestinal function, regulate glucose and amino acid metabolism, and ensure nutrient absorption.

Studies have shown that betaine regulates lipid metabolism via multiple mechanisms. Specifically, the mRNA levels of FASN directly affect the rate of triacylglycerol synthesis, and acetyl coenzyme A carboxylase (ACC) is the rate‐limiting enzyme in fatty acid synthesis. Furthermore, the activity of hormone‐sensitive esterase and lipoprotein esterase, as well as their corresponding gene expression, determines the level and activity of enzymatic proteins. Moreover, adipose tissue influences lipid synthesis and catabolism by regulating these key enzymes of lipid metabolism (Wei et al., 2012). In the present study, the pathways associated with lipid metabolism in the duodenal mucosa were as follows: non‐alcoholic fatty liver disease, AMPK signalling pathway, thermogenesis, fatty acid biosynthesis, fatty acid metabolism, unsaturated fatty acid biosynthesis and linoleic acid metabolism. Moreover, most of the DEGs enriched in lipid‐related pathways were significantly downregulated, such as sterol coenzyme A desaturase, ACACA, long‐chain fatty acid elongase 7 and FASN. Of these, ACACA is involved in the conversion of acetyl coenzyme A to malonyl coenzyme A in the tricarboxylic acid cycle, and FASN is the rate‐limiting enzyme for lipid synthesis. Based on the transcriptomic pathway, we found that the genes associated with these two enzymes were significantly downregulated. Reportedly, ACC exists in two main isoforms in animals: ACC1 and ACC2. These two forms are reciprocal isoenzymes, with ACC1 being mainly present in the cytoplasm, being encoded by ACACA, and being highly expressed in tissues that are active sites of lipid synthesis, such as the liver and adipose and mammary glands (Ryu et al., 2005). ACC1 primarily catalyses the conversion of acetyl coenzyme A to malonate monoacyl coenzyme A, which provides the substrate for the next step of FASN‐catalysed endogenous fatty acid synthesis.

## 5 CONCLUSION

Diets supplemented with 1500 mg/kg GAA and 600 mg/kg RPB increased ADG (from 1 to 30 days of experimental period) and apparent digestibility of EE and phosphorus, and GAA and creatine levels in small intestinal mucosal tissues in sheep. Supplemental feeding of RPB on top of GAA in sheep diets may thus promote sheep growth and development by improving the body's energy, amino acid, glucose and lipid metabolism capacity. This occurs by regulating the high expression of solute transporter protein genes, ATPase genes and genes implicated in pathways related to glucolipid metabolism and by promoting creatine synthesis through methyl metabolism that acts as a methyl donor.

## AUTHOR CONTRIBUTIONS

*Conceptualization, methodology, investigation, data curation, writing‐original draft and editing*: Chen Ma. *Conceptualization, methodology, writing, data curation, formal analysis, validation and editing*: Mireguli Yimamu. *Software, formal analysis and data curation*: Shiqi Zhang. *Investigation and editing*: Ali Mujtaba Shah. *Resources, investigation and data curation*: Hao Yang. *Software and data curation*: Wenjie Cai, Chaonan Li and Xuejie Lu. *Methodology and formal analysis*: Fengming Li. *Conceptualization, methodology, validation, writing‐review and supervision*: Kailun Yang.

## CONFLICT OF INTEREST STATEMENT

The authors declare no conflicts of interest.

### ETHICS STATEMENT

All experimental procedures involving animals were approved by the Animal Welfare and Ethics Committee of Xinjiang Agricultural University (animal protocol number: 2020024).

### PEER REVIEW

The peer review history for this paper is available at https://www.webofscience.com/api/gateway/wos/peer-review/10.1002/vms3.1470.

## Supporting information

Supporting Information

## Data Availability

The raw sequencing data from the current study have been deposited in the NCBI database with the accession number PRJNA1076840. Another data are available from the corresponding author.
