# Supplementary material for: Effects of guanidino acetic acid and betaine supplementation on growth, dietary nutrient digestion and intestinal creatine metabolism in sheep
Source: Vet Med Sci. 2024 Jun 24;10(4):e1470. doi: 10.1002/vms3.1470 (PMC11196381; doi:10.1002/vms3.1470)
Supplement: Supplementary file 1 — Supporting Information [file VMS3-10-e1470-s001.docx]

Supplementary Information

Effects of guanidineacetic acid and betaine supplementation on growth, dietary nutrient digestion, and intestinal creatine metabolism in sheep

**Table S1. Specifications of primers used in real-time fluorescence quantitative polymerase chain reaction**

| Gene name | Sequences of primers (5’-3’) | Product size/bp | GenBank ID |
| --- | --- | --- | --- |
| *SI* | RP: AAATGCTGGACCCCACAGAA | 89 | 101102774 |
|  | FP: GCACTGTTATCCGACCCCTT |  |  |
| *LOC101109677* | RP: GGTACTCAGGGTCTGGTGT | 147 | 101109677 |
|  | FP: ACTCAGATGCCGACCTGT |  |  |
| *GCK* | RP: GCCCTTGGTCCAGTTGAGAA | 170 | 101117412 |
|  | FP: CCATGACGGGTACTGCTGAG |  |  |
| *ACACA* | RP: GCTACGTGAGTCATGCCG | 70 | 443186 |
|  | FP: GGAACATCCCCACGCTAAA |  |  |
| *FASN* | RP:GGGAATACAGTTGGCCGTCA | 121 | 100170327 |
|  | FP:CTCAGTGGGCTCCTCAACTC |  |  |
| *DHFR* | RP:AGCTCCCTTTGGAGGTTC | 104 | 101121421 |
|  | FP:TGGGTAGGAAGACCTGGTT |  |  |
| *GAPDH* | RP: CCGCATCCCTGAGACAAGAT | 220 | 443005 |
|  | FP: CAAACATGGTGGTGATGTCCG |  |  |

FP: forward primer; RP: reverse primer.

**Table S2. RNA quality parameters in the intestinal mucosa**

| Group | Sample name | Concentration(ng/μL) | Volume (μL) | Total content (μg) | Integrity value | Test grade |
| --- | --- | --- | --- | --- | --- | --- |
| Test group Ⅰ | SENM-1 | 1155 | 55 | 63.525 | 6.7 | A |
|  | SENM-2 | 1720 | 55 | 94.6 | 7.1 | A |
|  | SENM-3 | 1109 | 55 | 60.995 | 7 | A |
|  | SENM-4 | 2466 | 55 | 135.63 | 8.5 | A |
| Test group Ⅱ | SENM-5 | 1748 | 55 | 96.14 | 5.7 | A |
|  | SENM-6 | 566 | 55 | 31.13 | 9.7 | A |
|  | SENM-7 | 2127 | 55 | 116.985 | 7.1 | A |
|  | SENM-8 | 786 | 45 | 35.37 | 10 | A |
| Test group Ⅰ | KNM-1 | 884 | 55 | 48.62 | 8.3 | A |
|  | KNM-2 | 798 | 55 | 43.89 | 10 | A |
|  | KNM-3 | 1950 | 55 | 107.25 | 9.5 | A |
|  | KNM-4 | 2104 | 45 | 94.68 | 5.1 | A |
| Test group Ⅱ | KNM-5 | 905 | 35 | 31.675 | 4.9 | A |
|  | KNM-6 | 1467 | 55 | 80.685 | 10 | A |
|  | KNM-7 | 891 | 55 | 49.005 | 10 | A |
|  | KNM-8 | 991 | 35 | 34.685 | 5.7 | A |

Note: SENM1–8 are duodenal mucosal samples, KNM1–8 are jejunal mucosa samples. The same as below.

**Table S3. Sequence quality and arrangement of intestinal mucosal samples in each group**

| Group | Sample name | reads  Raw reads | reads  Clean reads | Clean bases (G) | Error rate | Phred>20  Q20 (%) | Phred>30  Q30 (%) | GC (%) |
| --- | --- | --- | --- | --- | --- | --- | --- | --- |
| Test group Ⅰ | SENM-1 | 46915016 | 45948430 | 6.89 | 0.03 | 96.98 | 91.35 | 51.56 |
|  | SENM-2 | 46573886 | 45632916 | 6.84 | 0.03 | 96.80 | 90.99 | 52.02 |
|  | SENM-3 | 49978750 | 48747242 | 7.31 | 0.03 | 97.05 | 91.52 | 50.93 |
|  | SENM-4 | 47151498 | 46064116 | 6.91 | 0.03 | 96.99 | 91.33 | 51.40 |
| Test group Ⅱ | SENM-5 | 47016534 | 45988626 | 6.90 | 0.03 | 97.00 | 91.46 | 53.39 |
|  | SENM-6 | 48239066 | 46787418 | 7.02 | 0.03 | 97.18 | 91.87 | 50.71 |
|  | SENM-7 | 47987004 | 46819038 | 7.02 | 0.03 | 96.93 | 91.29 | 52.43 |
|  | SENM-8 | 45239388 | 44282968 | 6.64 | 0.03 | 96.79 | 90.97 | 51.11 |
| Test group Ⅰ | KNM-1 | 46261002 | 44952912 | 6.74 | 0.03 | 97.00 | 91.39 | 51.04 |
|  | KNM-2 | 46596776 | 45716910 | 6.86 | 0.03 | 96.98 | 91.38 | 52.21 |
|  | KNM-3 | 53719596 | 52203008 | 7.83 | 0.02 | 98.14 | 94.5 | 51.37 |
|  | KNM-4 | 54290892 | 52811076 | 7.92 | 0.02 | 98.08 | 94.35 | 50.80 |
| Test group Ⅱ | KNM-5 | 47993610 | 47061770 | 7.06 | 0.03 | 97.14 | 91.64 | 51.17 |
|  | KNM-6 | 48900034 | 47981528 | 7.20 | 0.03 | 96.74 | 90.85 | 50.84 |
|  | KNM-7 | 49442150 | 48278252 | 7.24 | 0.03 | 96.93 | 91.27 | 51.45 |
|  | KNM-8 | 49257732 | 48028112 | 7.20 | 0.03 | 97.21 | 91.95 | 50.89 |

Note: SENM1–8 are duodenal mucosal samples, KNM1–8 are jejunal mucosa samples.

**Table S4. Comparison rate statistical table of intestinal mucosa samples**

| Group | Sample name | Exon | Intron | Total map | Unique map | Multi map |
| --- | --- | --- | --- | --- | --- | --- |
| Test group Ⅰ | SENM-1 | 5646459806  (87.09%) | 516680540  (7.97%) | 43352336  (94.35%) | 39397247  (85.74%) | 3955089  (8.61%) |
|  | SENM-2 | 5499408327  (85.49%) | 647528806  (10.07%) | 43015974  (94.27%) | 39282603  (86.08%) | 3733371  (8.18%) |
|  | SENM-3 | 5542922606  (80.99%) | 916433419  (13.39%) | 45770165  (93.89%) | 42061749  (86.29%) | 3708416  (7.61%) |
|  | SENM-4 | 5602105203  (85.94%) | 606267788  (9.30%) | 43580945  (94.61%) | 39702837  (86.19%) | 3878108  (8.42%) |
| Test group Ⅱ | SENM-5 | 5527249640  (86.57%) | 563161519  (8.82%) | 42704128  (92.86%) | 39263919  (85.38%) | 3440209  (7.48%) |
|  | SENM-6 | 5909426000  (89.41%) | 363683240  (5.50%) | 44203342  (94.48%) | 38605153  (82.51%) | 5598189  (11.97%) |
|  | SENM-7 | 5756371470  (87.40%) | 531673033  (8.07%) | 44047111  (94.08%) | 40109163  (85.67%) | 3937948  (8.41%) |
|  | SENM-8 | 5349913338  (85.71%) | 580898481  (9.31%) | 41740106  (94.26%) | 37269464  (84.16%) | 4470642  (10.10%) |
| Test group Ⅰ | KNM-1 | 5540903919  (86.79%) | 548512227  (8.59%) | 42687633  (94.96%) | 38538962  (85.73%) | 4148671  (9.23%) |
|  | KNM-2 | 5664680212  (88.35%) | 435891596  (6.80%) | 42871288  (93.78%) | 39554208  (86.52%) | 3317080  (7.26%) |
|  | KNM-3 | 6108577214  (82.13%) | 939744116  (12.63%) | 49727716  (95.26%) | 45848823  (87.83%) | 3878893  (7.43%) |
|  | KNM-4 | 6512868946  (86.10%) | 694733456  (9.18%) | 50560966  (95.74%) | 44949582  (85.11%) | 5611384  (10.63%) |
| Test group Ⅱ | KNM-5 | 5665878424  (84.98%) | 662627021  (9.94%) | 44580229  (94.73%) | 40996416  (87.11%) | 3583813  (7.62%) |
|  | KNM-6 | 5962038962  (87.60%) | 498170009  (7.32%) | 45509937  (94.85%) | 40435853  (84.27%) | 5074084  (10.58%) |
|  | KNM-7 | 5713435272  (83.43%) | 766807888  (11.20%) | 45794121  (94.85%) | 42379867  (87.78%) | 3414254  (7.07%) |
|  | KNM-8 | 5793788371  (85.69%) | 627335004  (9.28%) | 45204479  (94.12%) | 40689960  (84.72%) | 4514519  (9.40%) |

Note: SENM1–8 are duodenal mucosal samples, KNM1–8 are jejunal mucosa samples.
